# Macrophages in Autoimmune Liver Diseases: From Immune Homeostasis to Precision-Targeted Therapy

**DOI:** 10.3390/biomedicines13102520

**Published:** 2025-10-16

**Authors:** Tianfu Liu, Yizhe Wang, Yichen Huang, Rui Zhao, Haili Shen

**Affiliations:** 1 Department of Hepatology, The Second Hospital & Clinical Medical School, Lanzhou University, Lanzhou 730030, China; drliutf@126.com (T.L.);; 2 Department of Respiratory and Critical Care Medicine, The First People Hospital of Lanzhou, Lanzhou 730050, China; 3 The Second Clinical Medical College, Lanzhou University, Lanzhou 730030, China; 4 Department of Rheumatology, The Second Hospital & Clinical Medical School, Lanzhou University, Lanzhou 730030, China

**Keywords:** autoimmune liver diseases, macrophages, programmed cell death, biomarkers, targeted therapy

## Abstract

Autoimmune liver diseases (AILDs) represent a diverse spectrum of chronic inflammatory conditions characterized primarily by compromised hepatic immune tolerance, including autoimmune hepatitis (AIH), primary biliary cholangitis (PBC), and primary sclerosing cholangitis (PSC). Recent evidence positions macrophages as pivotal players in AILDs pathogenesis, attributable to their multifaceted roles in inflammation amplification, immune regulation, and fibrogenesis. In the context of AILDs, macrophages exhibit marked polarization imbalance, increased recruitment of monocytes, and impaired clearance of apoptotic cells. Through complex interactions with T lymphocytes and hepatic stellate cells, macrophages orchestrate a pathological milieu promoting inflammation and fibrosis. Notably, diverse programmed cell death (PCD) modalities—autophagy, necroptosis, pyroptosis, and ferroptosis—not only determine macrophage survival and functional phenotype but also significantly impact cytokine release, phenotypic plasticity, and the trajectory of immunopathological progression. This review synthesizes current understandings of macrophage-driven immunoregulatory mechanisms in AILDs, characterizes the regulatory attributes of various macrophage-related PCD processes, and evaluates their relevance in experimental disease models. Furthermore, we highlight recent advancements in biomarker identification and targeted therapeutic strategies. Comprehensive elucidation of the interplay between macrophage immunological activity and programmed cell death pathways promises to inform novel, personalized therapeutic approaches for patients with AILDs.

## 1. Introduction

Autoimmune liver diseases (AILDs) constitute a diverse group of chronic inflammatory disorders characterized primarily by impaired hepatic immune tolerance, including autoimmune hepatitis (AIH), primary biliary cholangitis (PBC), and primary sclerosing cholangitis (PSC). AIH predominantly affects hepatocytes, leading to hallmark interface hepatitis; PBC chiefly targets interlobular bile duct epithelial cells, resulting in progressive ductular injury and potential obliteration; PSC is defined by inflammatory and fibrotic involvement of both intra- and extrahepatic bile ducts, ultimately causing bile duct obstruction. Collectively, these conditions share fundamental pathological characteristics such as chronic inflammation and progressive fibrosis, often culminating in cirrhosis and subsequent liver failure [1,2,3]. Despite recent advances, the precise pathogenesis of AILDs remains incompletely understood, hindering the identification of highly specific diagnostic and prognostic biomarkers and effective therapeutic interventions, thus substantially impacting patients’ quality of life. The global incidence of AILDs has demonstrated a steady upward trend, currently estimated at approximately 1–2 new cases per 100,000 individuals per year, posing significant public health challenges [4].

**Figure 1:**
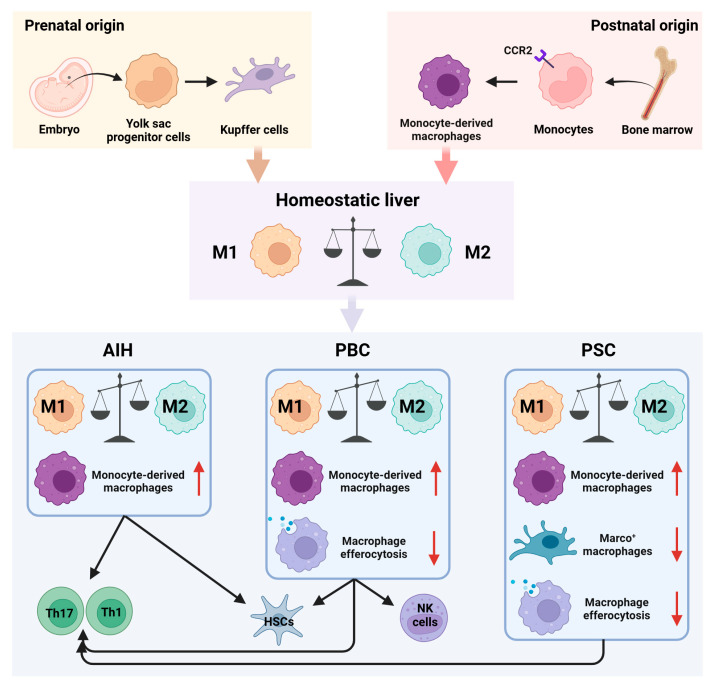
Role of macrophages in immune homeostasis and disease progression in AILDs. Hepatic macrophages primarily consist of resident Kupffer cells and monocyte-derived macrophages (MoMFs). Under physiological conditions, pro-inflammatory (M1) and anti-inflammatory (M2) macrophages maintain a dynamic equilibrium within the liver. However, in AILDs, disrupted macrophage polarization, excessive MoMFs recruitment, decreased numbers of Marco^+^ immunosuppressive macrophages, and impaired clearance of apoptotic cells collectively perturb hepatic immune homeostasis. Moreover, interactions between macrophages and other immune cells, including T cells, natural killer (NK) cells, and hepatic stellate cells (HSCs), further aggravate immune dysregulation and tissue injury characteristic of AILDs.

Within the hepatic environment, macrophages constitute the predominant innate immune cell population, accounting for about 20% of non-parenchymal cells in healthy liver tissue [5]. These macrophages play integral roles in immunological surveillance, tissue remodeling, and homeostatic maintenance. Liver macrophages are classified principally as resident Kupffer cells, derived from embryonic yolk-sac progenitors, and monocyte-derived macrophages (MoMFs), originating from circulating monocytes (Figure 1). Kupffer cells function predominantly as immune sentinels, maintaining hepatic tolerance by recognizing and eliminating invading pathogens, whereas MoMFs, responsive to inflammatory cues, orchestrate local inflammatory responses and mediate tissue repair processes [6,7]. Moreover, macrophages exhibit significant functional plasticity, dynamically transitioning into either pro-inflammatory (M1) or anti-inflammatory (M2) phenotypes, modulated by local microenvironmental signals [8]. Mounting evidence implicates dysregulated macrophage polarization and functional impairment as critical factors in the pathogenesis of chronic inflammation, cholangiopathy, and hepatic fibrosis associated with AILDs, underscoring their pivotal roles in disease progression [9,10,11].

Programmed cell death (PCD), encompassing apoptosis, autophagy, necroptosis, pyroptosis, and ferroptosis, significantly influences the pathogenesis, progression, and resolution of various hepatic disorders, profoundly affecting disease severity and clinical outcomes. Notably, macrophage-associated PCD pathways may intricately influence macrophage polarization and thereby modulate disease trajectories. However, the precise mechanisms underlying these interactions remain unclear and necessitate further investigation [12,13]. The relationship between macrophage PCD and immune regulatory functions, including phenotypic shifts, represents a critical area of current research interest in the context of AILDs [14,15].

This review comprehensively addresses recent developments in understanding macrophage-mediated immune regulation, elucidates macrophage-associated PCD mechanisms, evaluates emerging diagnostic and prognostic biomarkers, and examines macrophage-targeted therapeutic strategies in AILDs. Additionally, we outline potential directions for future research, aimed at advancing personalized diagnostics and therapeutics for AILDs.

## 2. Role of Macrophages in Immune Homeostasis and Disease Progression in AILDs

Macrophages, key innate immune cells, play crucial roles in modulating immune homeostasis and driving disease progression in AILDs. Through dynamic polarization between pro-inflammatory (M1) and anti-inflammatory (M2) phenotypes, macrophages significantly influence the hepatic immune milieu, thereby affecting adaptive immune cell activation and exacerbating autoimmune responses and tissue injury (Figure 1). Clarifying macrophage-specific immune mechanisms across different subtypes of AILDs is thus critical for understanding disease pathogenesis and identifying novel biomarkers and therapeutic targets.

### 2.1. Autoimmune Hepatitis (AIH)

Macrophages exert a central role in AIH, characterized by marked imbalance between pro-inflammatory and anti-inflammatory polarization. In AIH, pro-inflammatory M1 macrophages (CD86^+^NOS2^+^) are significantly elevated and positively correlate with inflammatory severity. Conversely, although anti-inflammatory M2 macrophages (CD206^+^Arg1^+^) increase numerically, their function is impaired, limiting their ability to effectively resolve inflammation [16]. Furthermore, MoMFs prominently infiltrate portal tracts and inflammatory interfaces within AIH liver tissues, creating a robust pro-inflammatory microenvironment [11,17]. The excessive recruitment and activation of these macrophages primarily depend on chemokine CCL2 signaling, which facilitates monocyte differentiation into M1 macrophages [11,18]. Elevated hepatic β-arrestin2 expression further promotes macrophage polarization via the ERK/p38 MAPK pathway, intensifying local inflammation [19].

Macrophages also contribute to AIH pathogenesis by modulating Th17/Treg cell balance. M1 macrophages release inflammatory cytokines such as interleukin-6 (IL-6), tumor necrosis factor-alpha (TNF-α), and IL-1β, thus enhancing Th17 cell expansion and inhibiting regulatory T-cell (Treg) differentiation, further exacerbating immune dysregulation [20]. Increased EZH2 expression, mediated by H3K27me3 histone modification, promotes M1 macrophage polarization, aggravating the Th1/Th2 imbalance [21]. Additionally, chronic inflammation activates the Kupffer cell–hepatic stellate cell (HSC) axis and accelerates fibrogenesis. Transmission electron microscopy of pediatric AIH livers revealed activated Kupffer cells in direct apposition to transitional HSCs, forming dense intercellular junctions (punctate desmosome-like contacts), suggesting that cell–cell adhesion provides a structural basis for their profibrotic crosstalk [22]. Paracrine TGF-β/PDGF and IL-1/TNF signaling from Kupffer cells can initiate and sustain the fibrogenic program of HSCs, whereas the heightened TLR4 sensitivity of HSCs drives chemokine-mediated recruitment of macrophages, establishing a self-reinforcing inflammatory–fibrotic loop [23,24,25]. Collectively, aberrant macrophage polarization and excessive recruitment in AIH not only fuel intrahepatic inflammation but also perpetuate Th1/Th17 disequilibrium and engage the Kupffer–HSC axis, thereby accelerating fibrosis.

### 2.2. Primary Biliary Cholangitis (PBC)

Macrophages are pivotal in the pathogenesis of PBC, significantly influencing cholangiocyte injury, immune activation, and fibrotic progression. Patients with PBC exhibit elevated peripheral blood levels of CD14^+^CD16^+^ monocytes-macrophage precursors-particularly CD14lowCD16^+^ subsets, correlating positively with hepatic damage markers and Th1 cell activity, implicating them in inflammatory responses [26]. Hepatic tissues from PBC patients demonstrate enriched MoMFs populations around bile ducts, which secrete IL-23, activating the IL-23/IL-17 axis and exacerbating cholangiocyte injury [27]. Guillot et al. reported substantial accumulation of IBA1^+^ macrophages around injured bile ducts, directly amplifying local inflammation through interactions with bile duct epithelial cells (BECs) [28]. Moreover, immunogenic PDC-E2 apoptotic bodies released by injured BECs interact with anti-mitochondrial antibodies (AMA), triggering macrophages to release IL-6 and TNF-α [29,30]. Impaired macrophage-mediated clearance of apoptotic BECs, caused by increased expression of transcription factor Arid3a that suppresses the Mertk receptor, further exacerbates inflammation in PBC [10].

Macrophages also facilitate PBC pathology through interactions with other immune cell types. Granuloma formation in early-stage PBC primarily results from activated macrophages and dendritic cells accumulating at portal sites, amplifying local inflammation [31]. Kupffer cells activate natural killer (NK) cells via pro-inflammatory cytokines, enhancing BECs stress, apoptosis, and mitochondrial dysfunction, driving immune-inflammatory progression [32]. It is worth noting that, in human PBC livers, causal evidence for direct KC–HSC interactions as drivers of fibrogenesis remains lacking. Nevertheless, studies indicate that macrophage-derived cathepsin S (CTSS) can remodel the extracellular matrix and liberate bioactive matrikines, thereby indirectly promoting HSC activation and contributing to cholestasis-associated fibrosis [23,33]. Clinically, serum CTSS concentrations are elevated in patients with PBC and show positive correlations with alkaline phosphatase (ALP) and γ-glutamyl transferase (GGT), suggesting a link between CTSS and the burden of cholangitis/fibrosis [34]. Thus, macrophages aggravate cholangiopathy and fibrosis in PBC through mechanisms involving inflammatory amplification, impaired apoptotic cell clearance, and CTSS-mediated fibrogenesis.

### 2.3. Primary Sclerosing Cholangitis (PSC)

The pathological progression of PSC involves profound remodeling of the hepatic immune microenvironment, prominently influenced by macrophage dysregulation. Liver tissues from PSC patients show substantial MoMFs accumulation around bile ducts coupled with reduced Kupffer cell populations [28,35]. Particularly diminished are immunosuppressive Marco^+^ Kupffer cells, which produce IL-10 and maintain hepatic immune tolerance, intensifying local immune dysregulation [9]. Cholangiocytes secrete chemokines CCL2 and IL-8, driving CCR2^+^ monocyte recruitment and differentiation into pro-inflammatory MoMFs [36]. Upregulated expression of POU6F1 and lncRNA H19 in PSC cholangiocytes enhances CCL2 transcription directly and indirectly induces Kupffer cell polarization toward the M1 phenotype via exosomal signaling, further augmenting inflammation [37,38]. Genome-wide association studies have identified MST-1, a critical non-HLA susceptibility gene, whose mutation restricts macrophage transition to the anti-inflammatory M2 phenotype, thus perpetuating chronic inflammation in PSC [39]. Additionally, disrupted bile acid signaling, characterized by reduced hepatic farnesoid X receptor (FXR) expression, facilitates excessive NOD-like receptor family pyrin domain containing 3 (NLRP3) inflammasome activation, promoting Th1/Th17 responses and exacerbating cholangiopathy [40]. As in PBC, increased hepatic Arid3a expression impairs macrophage-mediated apoptotic cholangiocyte clearance, further aggravating inflammation [10].

Persistent inflammation results in extensive accumulation of osteopontin^+^ macrophages in fibrotic peri-biliary regions, facilitating interactions between cholangiocytes and fibroblasts via the CXCL12–CXCR4 axis, thereby accelerating fibrosis [41]. Macrophages also regulate hepatic progenitor cell activation through the Notch signaling pathway, disrupting normal cholangiocyte repair and exacerbating ductal architectural distortion and fibrosis [42]. Thus, macrophage subset imbalances, aberrant activation, and impaired apoptotic clearance collectively create a pro-inflammatory, pro-fibrotic microenvironment, significantly advancing PSC pathology.

## 3. Role of Programmed Cell Death in Macrophages in AILDs

**Figure 2:**
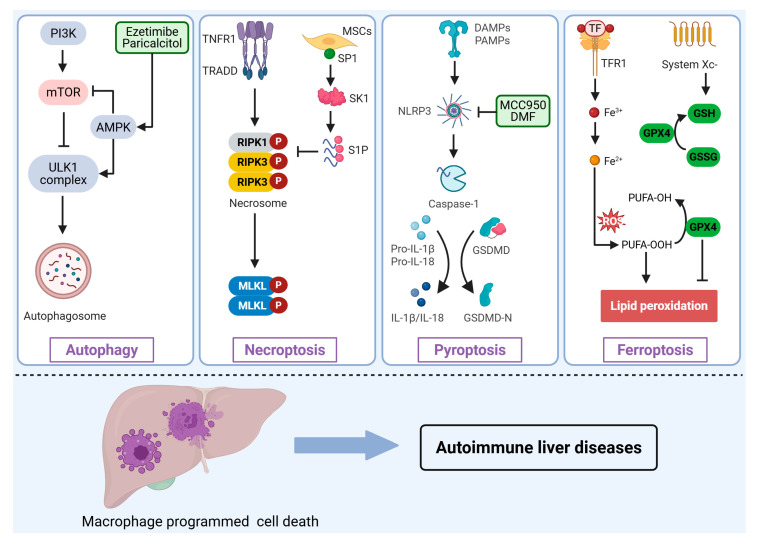
Involvement of macrophage programmed cell death in the pathogenesis of AILDs. Various stimuli or signals induce distinct forms of programmed cell death in macrophages, including autophagy, necroptosis, pyroptosis, and ferroptosis, thus regulating the initiation and progression of AILDs. PI3K, Phosphoinositide 3-Kinase; mTOR, Mechanistic target of rapamycin; AMPK, AMP-activated protein kinase; ULK1, Unc-51 kike autophagy activating kinase 1; TNFR1, tumor necrosis factor receptor 1; TRADD, TNF receptor-associated death domain protein; RIPK, receptor-interacting protein kinase; MLKL, Mixed lineage kinase domain-like protein; MSCs, Mesenchymal Stem Cells; SP1, specificity protein 1; SK1, sphingosine kinase 1; S1P, sphingosine-1-Phosphate; NLRP3, NOD-like receptor family pyrin domain containing 3; DMF, dimethyl fumarate; GSDMD, Gasdermin D; TF, Transferrin; TFR1, Transferrin receptor 1; GPX4, Glutathione peroxidase 4; GSH, Glutathione; GSSG, Glutathione; ROS, Reactive oxygen species; PUFA-OH, Polyunsaturated fatty acid hydroxide; PUFA-OOH, Polyunsaturated fatty acid hydroperoxide.

Dysregulated PCD pathways profoundly influence macrophage survival, activation, and polarization, thereby reshaping the hepatic immune microenvironment and driving inflammatory and fibrotic processes. Although apoptosis is historically the most studied form of PCD, direct evidence and clinically actionable targets remain relatively limited in the context of AILDs. By contrast, PCD modalities with overt pro-inflammatory signatures-autophagy, necroptosis, pyroptosis, and ferroptosis-have emerged as key regulators of macrophage phenotypic switching, inflammatory mediator release, and tissue injury (Figure 2). A deeper mechanistic understanding of these pathways could provide novel insights into AILDs pathogenesis and inform potential therapeutic targets.

### 3.1. Macrophage Apoptosis

Apoptosis is governed by extrinsic cues (e.g., Fas/FasL, TNF/TNFR1) and the intrinsic mitochondrial pathway, culminating in cell clearance through a caspase cascade. In hepatic inflammation and fibrosis, macrophage apoptosis can both curb the pool of hyperactivated cells and dampen pro-inflammatory mediator release; in parallel, exposure of “eat-me” signals (such as phosphatidylserine) facilitates efferocytosis, thereby hastening resolution of inflammation and promoting tissue repair [43]. Direct human evidence specific to AILDs remains scarce, yet several mechanistic leads are informative. Under AIH-relevant environmental exposure, metabolites of trichloroethylene directly trigger Kupffer cell apoptosis and impair phagocytic clearance, potentially undermining immune tolerance and amplifying autoimmunity [44]. In fatty liver disease models, M2-polarized Kupffer cells selectively induce apoptosis of M1 counterparts via an IL-10/arginase axis, lowering inflammatory burden and suggesting that “M2-driven M1 apoptosis” helps maintain lineage equilibrium [45]. In fibrotic livers from both humans and mice, CHI3L1 is highly expressed in hepatic macrophages and suppresses their apoptosis; genetic loss of CHI3L1 enhances macrophage apoptosis and attenuates fibrosis, implying that judicious promotion of macrophage apoptosis may confer anti-fibrotic benefit [46]. Within AILD contexts, it is imperative to delineate the determinants and kinetics of macrophage apoptosis and to causally anchor its impact on inflammatory resolution and fibrotic remodeling with robust biomarkers.

### 3.2. Macrophage Autophagy

Autophagy is a highly conserved lysosome-mediated catabolic pathway that maintains cellular homeostasis by degrading and recycling damaged proteins and organelles [47]. Macrophage autophagy is intricately linked to polarization; impaired autophagic activity preferentially promotes differentiation toward the pro-inflammatory M1 phenotype, exacerbating inflammation and chronic liver injury [48,49]. Conversely, enhanced autophagy promotes anti-inflammatory M2 polarization, thus limiting inflammation and facilitating tissue repair. Consequently, autophagy acts as a pivotal negative feedback mechanism modulating macrophage-mediated inflammatory responses. The autophagic process is tightly regulated by multiple signaling pathways, notably the mechanistic target of rapamycin (mTOR) and AMP-activated protein kinase (AMPK) pathways [50]. Specifically, the PI3K/AKT pathway suppresses autophagy via mTOR complex 1 (mTORC1) activation, whereas energy deficit conditions activate AMPK, which inhibits mTORC1 and directly initiates autophagy via the Unc-51 kike autophagy activating kinase 1 (ULK1) complex [51,52]. Activation of AMPK signaling enhances macrophage autophagy, thereby promoting M2 polarization and mitigating inflammatory responses [53].

Experimental models of AILDs underscore the importance of macrophage autophagy in disease progression. For instance, in concanavalin A (ConA)-induced AIH, Atg7 knockout mice exhibit heightened macrophage activation, pro-inflammatory cytokine secretion, and liver injury [54]. Similarly, reduced macrophage autophagy correlates with excessive NLRP3 inflammasome activation in cholestatic liver injury models (bile duct ligation, BDL) resembling PBC and PSC. Activation of macrophage autophagy via the vitamin D receptor pathway reduces inflammasome-driven IL-1β production, alleviating hepatic inflammation [55]. Thus, therapeutic modulation of macrophage autophagy represents a promising clinical strategy in AILDs management.

### 3.3. Macrophage Necroptosis

Necroptosis is a regulated, pro-inflammatory form of necrosis mediated predominantly by the receptor-interacting protein kinase 1 (RIPK1), RIPK3, and mixed lineage kinase domain-like (MLKL) signaling axis [56]. Unlike apoptosis, necroptosis involves disruption of membrane integrity and the release of damage-associated molecular patterns (DAMPs), such as HMGB1, ATP, and heat-shock proteins, thereby amplifying local inflammation [57,58].

Accumulating evidence implicates macrophage necroptosis in AILD pathogenesis. Elevated RIPK3 expression in hepatic macrophages of AIH patients positively correlates with liver injury severity [59]. Additionally, disruption of the specificity protein 1 (Sp1)/sphingosine kinase-1 (SK1)/sphingosine-1-phosphate (S1P) signaling axis observed in AIH enhances necroptotic activity, thereby exacerbating inflammation [14]. Interestingly, pharmacological interventions such as broad-spectrum caspase inhibitors (e.g., zVAD) can divert macrophage cell death from apoptosis to necroptosis, paradoxically ameliorating liver damage in experimental AIH models [60]. In pediatric livers from patients with biliary atresia and in mice subjected to bile duct ligation, bile acid metabolites such as glycodeoxycholic acid (GDCA) trigger macrophage necroptosis via S1PR2-ZBP1-MLKL signaling, exacerbating inflammatory and fibrotic responses [61]. Furthermore, caspase-10 mutations identified in PBC may simultaneously activate necroptosis and pyroptosis in macrophages, whereas therapies with ursodeoxycholic acid or obeticholic acid can partially reverse these aberrant death pathways [15]. Thus, targeting macrophage necroptosis represents a potentially valuable therapeutic strategy in AILDs treatment.

### 3.4. Macrophage Pyroptosis

Pyroptosis is an inflammasome-mediated cell death pathway characterized by profound inflammatory effects [62]. Canonical pyroptosis involves activation of the NLRP3 inflammasome by pathogen-associated or damage-associated molecular patterns, subsequently activating caspase-1, which cleaves Gasdermin D (GSDMD). GSDMD cleavage induces pore formation, cellular lysis, and release of pro-inflammatory cytokines, notably IL-1β and IL-18 [63,64]. Non-canonical pyroptotic pathways mediated by caspase-3/GSDME or granzyme A/GSDMB axes similarly amplify inflammation [65,66]. Cytokines released during pyroptosis notably enhance Th17 differentiation and hepatic stellate cell activation, intensifying local inflammation and fibrosis [67,68,69].

Pyroptosis of macrophages is increasingly recognized as a critical factor in AILDs pathology. Enhanced NLRP3 inflammasome activation and macrophage pyroptosis have been demonstrated in ConA-induced AIH, where pharmacological inhibition of inflammasome components markedly attenuates hepatic injury [70]. Similarly, caspase-10 mutations in PBC intensify macrophage pyroptosis, promoting disease progression [15]. Intriguingly, GSDMD, the executor protein in pyroptosis, exhibits context-dependent roles; GSDMD-deficient mice paradoxically show exacerbated liver injury under certain inflammatory conditions, suggesting possible protective functions under specific pathophysiological contexts [71]. Collectively, macrophage pyroptosis emerges as a critical inflammatory amplification mechanism warranting further investigation.

### 3.5. Macrophage Ferroptosis

Ferroptosis is an iron-dependent form of cell death characterized by lipid peroxidation and subsequent membrane damage [72]. Macrophages, as central regulators of systemic iron metabolism, are particularly susceptible to ferroptotic cell death under inflammatory conditions, exacerbating tissue injury [73,74]. Cellular defenses against ferroptosis predominantly rely on glutathione peroxidase 4 (GPX4), which detoxifies lipid peroxides via glutathione-dependent mechanisms. GPX4 activity is reliant on glutathione synthesized through the cystine/glutamate antiporter system Xc^−^, constituting a crucial anti-ferroptotic pathway [75].

Emerging evidence highlights the potential involvement of macrophage ferroptosis in autoimmune conditions [76,77]. Animal models of AIH demonstrate increased hepatic iron accumulation, lipid peroxidation (elevated malondialdehyde levels), and diminished GPX4 and system Xc^−^ protein expression, indicative of ferroptosis activation [78]. Similarly, in S100-induced autoimmune liver injury, upregulated ferroptosis markers (ACSL4, COX2) alongside reduced GPX4 and ferritin heavy chain (FTH1) expression correlate with disease severity. Loss of GPX4 exacerbates hepatic damage, reinforcing its protective role [79]. Nevertheless, macrophage-specific ferroptosis pathways remain inadequately explored, and direct evidence connecting macrophage ferroptosis with macrophage polarization, cytokine secretion, and tissue injury in AILDs remains limited. Consequently, macrophage ferroptosis constitutes an emerging research area with significant therapeutic potential deserving comprehensive investigation.

## 4. Clinical Relevance of Macrophage-Derived Biomarkers in the Diagnosis and Prognosis of AILDs

The activation state and functional alterations of macrophages are intimately associated with the pathogenesis and progression of AILDs. Consequently, considerable research efforts have been directed toward identifying macrophage-specific biomarkers capable of enhancing diagnostic accuracy and prognostic prediction. Among these markers, macrophage surface receptors, notably CD163 and the mannose receptor (MR), serve as canonical indicators of the alternatively activated M2 macrophage phenotype, playing essential roles in heme clearance and the maintenance of immune homeostasis. Under inflammatory conditions, proteolytic cleavage of these membrane-bound receptors generates soluble forms (sCD163 and sMR) that enter systemic circulation, thereby reflecting the extent of macrophage activation. Thus, these soluble biomarkers have emerged as valuable tools for assessing disease activity and predicting clinical outcomes (Table 1).

In patients with AIH, elevated serum sCD163 levels correlate closely with disease activity, therapeutic responses, and the risk of relapse, showing marked reductions following successful immunosuppressive therapy. Hence, serum sCD163 represents a potentially useful biomarker for clinical monitoring in AIH [80]. Similarly, increased serum concentrations of sCD163 and sMR in patients with PBC have been associated with portal hypertension and progressive hepatic fibrosis. Notably, serum sMR levels exceeding 56.6 ng/mL independently predict disease progression and complication risk in this patient population [81]. Moreover, combining serum sCD163 and sMR measurements with established predictive tools such as the UK-PBC score significantly enhances prognostic accuracy [82]. Notably, elevated salivary sCD163 concentrations in PBC patients correlate with alterations in the oral microbiome, suggesting a potential mechanistic link between oral microbial dysbiosis, macrophage activation, and enhanced local or systemic inflammation, thereby providing novel insights into PBC pathogenesis [83]. In PSC, increased serum levels of sCD163 and sMR strongly correlate with disease severity, Mayo risk scores, and the likelihood of requiring liver transplantation [84]. Furthermore, elevated urinary sCD163 has demonstrated substantial diagnostic utility for PSC, with an area under the curve (AUC) of 0.733, highlighting its potential role in clinical diagnosis and differential diagnosis of PSC [85]. Although Arid3a is not conventionally considered a macrophage activation marker, its pivotal role as a transcriptional regulator of macrophage function merits attention. Elevated Arid3a expression in peripheral blood mononuclear cells (PBMCs) from patients with PBC and PSC positively correlates with liver injury markers, including ALP, GGT, and total bilirubin (TBil), indicating its potential utility as an indirect biomarker reflecting macrophage functionality [10]. Taken together, macrophage-derived biomarkers such as sCD163 and sMR hold significant clinical promise for the diagnosis, monitoring, and prognostication of AILDs. Future validation studies are warranted to firmly establish their roles in clinical practice, ultimately facilitating personalized therapeutic approaches.

## 5. Advances and Therapeutic Strategies Targeting Macrophages in AILDs

Macrophages have emerged as key therapeutic targets in AILDs owing to their pivotal roles in orchestrating inflammatory responses and mediating hepatic tissue injury and repair. Current research emphasizes precise modulation of macrophage polarization, recruitment, and PCD pathways as strategies for controlling pathological immune responses. This review systematically addresses recent advancements in macrophage-targeted therapies, focusing on polarization and recruitment modulation, intervention in programmed cell death, and the application of nanoparticle-based drug delivery systems.

### 5.1. Regulation of Macrophage Polarization and Recruitment

Macrophage polarization critically influences both inflammatory and tissue reparative processes within the liver. Classically activated M1 macrophages typically mediate pro-inflammatory effects, whereas alternatively activated M2 macrophages predominantly support anti-inflammatory responses and tissue repair. In patients with AILDs, the hepatic microenvironment favors M1 macrophage predominance, accompanied by substantial infiltration of MoMFs, perpetuating chronic inflammation. Consequently, strategies aimed at restoring macrophage polarization balance and mitigating excessive recruitment represent central therapeutic objectives.

Numerous pharmacologic agents have demonstrated efficacy in modulating macrophage polarization. For instance, pemetrexed, an NF-κB pathway inhibitor, attenuates M1 macrophage activation, effectively reducing liver injury in ConA-induced hepatitis models [86]. Similarly, allyl methyl disulfide suppresses M1 polarization and inhibits NLRP3 inflammasome activation, thereby ameliorating inflammatory responses in AIH [87]. Additionally, inhibitors targeting EZH2 or MARCO proteins, such as DZNep and polyguanine, respectively, enhance M2 polarization, showing therapeutic promise in experimental AIH [21,88]. Fibroblast growth factor 4 (FGF4), acting via the PI3K/Akt signaling pathway, also shifts macrophage polarization toward the M2 phenotype, significantly reducing inflammatory damage in ConA-induced hepatitis models; notably, this protective effect is lost upon macrophage depletion, underscoring the specificity of macrophage-mediated responses [16].

Targeting macrophage recruitment pathways, particularly the CCL2–CCR2 axis, represents another important therapeutic avenue. Cenicriviroc, a dual CCR2/CCR5 antagonist, effectively reduces hepatic infiltration of MoMFs, thereby ameliorating inflammation across various AILD models [11,36,89]. In α-naphthyl isothiocyanate (ANIT)-driven acute/chronic PSC mouse models, 18β-glycyrrhetinic acid suppresses POU6F1-dependent CCL2 transcription, thereby limiting the recruitment of CCR2^+^ MoMFs and attenuating periductal inflammation and fibrosis [37]. Enhancing macrophage clearance of apoptotic cholangiocytes by activation of the Mertk pathway or inhibition of Arid3a expression also emerges as a promising strategy to indirectly alleviate inflammation and restore hepatic homeostasis [10].

### 5.2. Targeting Macrophage PCD Pathways

Therapeutic interventions targeting macrophage PCD pathways offer a promising approach for precision immunoregulation in AILDs (Figure 2). Dysregulation of macrophage autophagy, necroptosis, and pyroptosis pathways has been closely linked to inflammatory activation and hepatic tissue injury.

Enhancing macrophage autophagy effectively reduces inflammation by preventing inflammasome hyperactivation. For instance, ezetimibe-induced hepatic macrophage autophagy significantly inhibits NLRP3 inflammasome activity, mitigating hepatic inflammation in experimental steatohepatitis models [90]. Similarly, the vitamin D receptor agonist paricalcitol enhances macrophage autophagy, reducing IL-1β secretion and indirectly attenuating pyroptotic inflammation in cholestatic liver diseases [55].

Necroptosis, mediated primarily through RIPK3 and MLKL pathways, has emerged as another viable therapeutic target. Restoration of SK1 expression through exogenous supplementation with SP1, as in mesenchymal stem cell therapy, inhibits macrophage necroptosis and reduces inflammation in severe AIH models [14]. Furthermore, macrophage-specific delivery of RIPK3-targeting siRNA via exosomes derived from M2 macrophages successfully attenuates necroptotic signaling, reducing hepatic inflammation and injury in AIH mouse models [20].

Pyroptosis, driven by the activation of NLRP3 inflammasomes, remains central to ongoing therapeutic research. MCC950, a selective NLRP3 inhibitor, exhibits potent anti-inflammatory and antifibrotic effects, promoting M2 polarization in macrophages [91]. Additionally, dimethyl fumarate (DMF) effectively suppresses NLRP3 inflammasome activity and reduces macrophage pyroptosis, significantly ameliorating liver injury in AIH experimental models [70]. Caspase-3 inhibitors, such as Ac-DMPD/DMLD-CMK, effectively block gasdermin E (GSDME)-mediated pyroptosis, suggesting potential therapeutic applications in cholestatic liver disease [92].

### 5.3. Nanoparticle-Based Macrophage-Targeted Delivery Systems

Nanoparticle-based drug delivery systems represent a promising therapeutic strategy, enabling precise targeting of macrophage populations within hepatic tissues and potentially enhancing therapeutic efficacy while minimizing systemic adverse effects.

Curdlan-decorated fullerene nanoparticles (Cur-F), which specifically target macrophages via the Dectin-1 receptor, significantly reduce hepatic MoMFs infiltration and inhibit NF-κB signaling, effectively alleviating inflammation in AIH models(ConA-induced mice) [93]. Glycosylated gold nanoparticles (Glyco-GNPs), selectively targeting CD206-expressing M2 macrophages, reduce M1 macrophage infiltration, promote M2 polarization, and attenuate periductal inflammation and fibrosis in PBC experimental models (ARE Del^−/−^ mice) [94]. In patient-derived PSC organoids and in the 3,5-diethoxycarbonyl-1,4-dihydrocollidine (DDC) diet-induced murine model of PSC, a ROS-responsive nanodelivery platform (MPPFTU@OCA) releases the FXR agonist obeticholic acid at lesional sites, thereby modulating bile acid metabolism and simultaneously suppressing macrophage-driven inflammation and programmed cell death [95].

Despite promising preclinical outcomes, significant challenges remain in translating nanoparticle-based therapies into clinical practice, including long-term safety concerns, immunogenicity, delivery efficacy, and scalability for clinical manufacturing. Further optimization of nanoparticle carrier materials and targeting mechanisms, alongside integration with advanced genetic and RNA interference technologies, will be crucial for advancing clinical translation and ultimately providing more precise and effective treatments for patients with autoimmune liver diseases.

In sum, approaches that target macrophages-spanning polarization/recruitment control, perturbation of programmed cell death, and nanocarrier-based delivery-have demonstrated value in substitute models and early-stage translation. Realizing prognostic benefit will nonetheless demand prospective, mechanistically anchored trials in AIH, PBC, and PSC, assessed against patient-relevant endpoints including reduced inflammatory activity, fibrosis regression, and transplant-free survival.

## 6. Conclusions and Future Perspective

This review has systematically summarized the central role of macrophages in the pathogenesis of autoimmune liver diseases, emphasizing their immunoregulatory functions across AIH, PBC, and PSC, and the underlying molecular mechanisms involving diverse programmed cell death pathways contributing to hepatic inflammation and fibrosis. Additionally, we have highlighted promising macrophage-derived biomarkers such as sCD163 and sMR, underscoring their potential diagnostic and prognostic tools. Recent advancements in macrophage-targeted therapeutic strategies—ranging from polarization modulation and recruitment inhibition to precise manipulation of programmed cell death pathways—underscore the shift toward multi-target and multi-mechanism approaches. Moreover, emerging nanoparticle-based drug delivery platforms offer feasible strategies to achieve highly selective and efficient macrophage-specific therapies.

Despite these advances, several important challenges remain. First, a deeper mechanistic understanding of macrophage polarization and functional heterogeneity is needed to clarify the distinct roles played by macrophage subsets at various stages of AILDs. Second, elucidating the complex interplay between distinct macrophage cell death pathways and their specific contributions to disease initiation and progression remains critical, necessitating further mechanistic investigation and validation in relevant animal models. Third, translating macrophage-targeted therapeutic strategies into clinical practice continues to face substantial hurdles, including challenges related to targeted delivery efficiency, long-term safety profiles, and individualized therapeutic responses.

Future research should prioritize several key objectives: first, systematically delineating the temporal dynamics and functional diversity of macrophage subsets throughout the course of AILDs progression; second, clarifying the interplay between macrophage polarization processes and programmed cell death mechanisms to provide an integrated understanding of disease pathogenesis; and third, integrating multi-omics methodologies with advanced nanoparticle-based targeting platforms to facilitate clinical translation. As our understanding deepens, macrophages may evolve from broadly defined immunomodulatory targets into precisely controlled therapeutic nodes, opening novel avenues toward individualized treatment strategies for patients with AILDs.

## Figures and Tables

**Table 1:** Summary of clinical studies on macrophage-related biomarkers in AILDs.

Disease	Biomarker(s)	Sample	Study Design/Sample Size	Primary Associations/Outcomes	References
AIH	sCD163	Serum	Single-center cross-sectional: *n* = 121	Disease activity, treatment response, relapse risk	[80]
PBC	sMR; sCD163	Serum	Single-center cohort: *n* = 77; median follow-up 4.4 years	Risk of disease progression and complications	[81]
PBC	sMR; sCD163	Serum	Multicenter cohort: *n* = 202; median follow-up 8.6 years	Long-term outcomes (liver-related death/liver transplantation)	[82]
PBC	sCD163	Saliva	Case–control: PBC *n* = 39, controls *n* = 37	Disease severity; inflammatory burden	[83]
PBC	CTSS	Serum	Case–control: PBC *n* = 32, healthy controls *n* = 27	Disease severity; association with cholestatic indices	[34]
PSC	sCD163; sMR	Serum	Two-center cohort: *n* = 297; follow-up ~5–8 years	Disease severity; long-term prognosis	[84]
PSC	sCD163	Urine	Case–control: PSC *n* = 21, healthy controls *n* = 18, other diseases *n* = 79	Differential diagnosis vs. other liver diseases	[85]

Abbreviations: AILDs, autoimmune liver diseases; AIH, autoimmune hepatitis; PBC, primary biliary cholangitis; PSC, primary sclerosing cholangitis; sCD163, soluble CD163; sMR, soluble mannose receptor; CTSS, cathepsin S.

## Data Availability

No new data were created or analyzed in this study. Data sharing is not applicable.
